# Chromatin structure profile data from DNS-seq: Differential nuclease sensitivity mapping of four reference tissues of B73 maize (*Zea mays* L)

**DOI:** 10.1016/j.dib.2018.08.015

**Published:** 2018-08-10

**Authors:** Zachary M. Turpin, Daniel L. Vera, Savannah D. Savadel, Pei-Yau Lung, Emily E. Wear, Leigh Mickelson-Young, William F. Thompson, Linda Hanley-Bowdoin, Jonathan H. Dennis, Jinfeng Zhang, Hank W. Bass

**Affiliations:** a Department of Biological Science, Florida State University, Tallahassee, FL 32306-4295, USA; b Center for Genomics and Personalized Medicine, Florida State University, Tallahassee, FL, USA; c Department of Statistics, Florida State University, Tallahassee, FL, 32306, USA; d Department of Plant and Microbial Biology, North Carolina State University, Raleigh 27606, USA

## Abstract

Presented here are data from Next-Generation Sequencing of differential micrococcal nuclease digestions of formaldehyde-crosslinked chromatin in selected tissues of maize (*Zea mays*) inbred line B73. Supplemental materials include a wet-bench protocol for making DNS-seq libraries, the DNS-seq data processing pipeline for producing genome browser tracks. This report also includes the peak-calling pipeline using the iSeg algorithm to segment positive and negative peaks from the DNS-seq difference profiles. The data repository for the sequence data is the NCBI SRA, BioProject Accession *PRJNA44570*8.

**Specifications Table**

**Table:** 

Subject area	Biology
More specific subject area	Plant Chromatin Structure Profiling using Nuclease Sensitivity Assay
Type of data	Next-Generation Sequencing Data
How data was acquired	NEBNext Ultra II DNA Library Prep
	Illumina HiSeq. 2500 (Paired-End 50 base reads)
Data format	Raw sequence non-interleaved.fastq files
Experimental factors	None, tissues were from normal (wild-type) plants
Experimental features	Formaldehyde-fixed nuclei were purified from maize tissues and subjected to light or heavy micrococcal nuclease digestion, followed by decrosslinking and purification of genomic DNA for NGS library preparation. Size-selected libraries were sequenced on the Illumina HiSeq. 2500 platform.
Data source location	Samples were from Tallahassee, FL, FSU research field (earshoot, endosperm), greenhouse (coleoptilar node), or from Raleigh NC, NCSU lab-grown (root-tip).
Data accessibility	Data is with this article and available at NCBI SRA repository under the Project Accession PRJNA445708 “Differential MNase Profiling of Select Zea Mays Tissues”
	https://www.ncbi.nlm.nih.gov/bioproject/PRJNA445708
Related research article	none

**Value of the data**

• These data provide a public resource for integrative and comparative analysis of chromatin structure in maize, a major crop and model organism.
• These data provide basic information about chromatin structure that could accelerate crop improvement strategies to address current and future challenges in food and biofuel production.
• These data define genome-wide differences in chromatin structure in four diverse tissues of maize.
• These data allow for discovery of so-called open chromatin linked to gene regulation.
• These data can be used for comparative analysis of MNase-seq to other chromatin accessibility assays such as ATAC-seq, DNaseI-seq, and FAIRE-seq.

## 1 Data

The sequence data are 50 bp paired-end reads from Illumina Hi-Seq. 2500 and the files are named as follows. *FRT1Ha_R1.fastq.gz* refers to *F*SU-grown 1 mm *R*oot *T*ips, Biological Replicate *1*, *H*eavy digest, technical replicate a, read 1. For each tissue, there are four sets of libraries, heavy and light digests, and their replicates. These digest pairs are later used to produce the "difference" files which capture the differential nuclease sensitivity, DNS. For comparison, the heavy digests alone are typical of conventional nucleosome occupancy mapping data, whereas the light digests are needed for the difference calculation.

In order to facilitate subsequent analysis, each data file (.fastq formatted) described in the NCBI SRA BioProject Accession *PRJNA445708* can be named using the above schema to capture uniquely identifying information about each sample in the file name.

## 2 Experimental design, materials, and methods

### 2.1 Development of DNS-seq to study maize chromatin structure

We have developed and refined Differential Nuclease Sensitivity (DNS) as a procedure for mapping nucleosome occupancy and open chromatin in maize [1], [2], [3]. Briefly, fixed nuclei are digested with a diffusible enzymatic probe, micrococcal nuclease (MNase, EC 3.1.31.1). Two digest conditions, “light” and “heavy” are employed and the resulting genomic DNA fragments are quantified by Next Generation Sequencing (NGS) to obtain the relative abundance of aligned fragments. The wet-bench protocol used to produce the DNS sequencing libraries is provided in Supplemental file 1. This protocol describes many tissue- and digestion condition- specific considerations for isolation of fixed nuclei, selection of appropriate MNase digestion conditions, and construction of Next-Generation Sequencing library pairs.

**Fig. 1:**
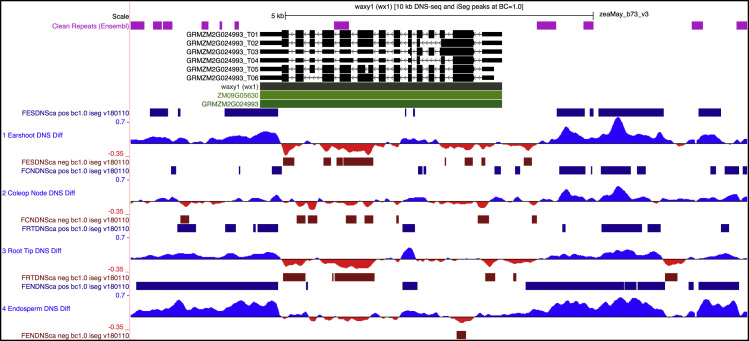
Browser screenshot from genomaize.org, a UCSC browser for maize B73. A screenshot showing a 10 kilobase pair region around the waxy1 (wx1) gene on chromosome 9. Repeat DNA (pink), gene models (black), gene identifiers (green) are shown above the DNS-seq profiles (blue/positive and red/negative difference value histograms) and their associated peak calls at a stringency of "BC=1.0" [4]. Track names for the DNS-seq profiles are shown at left and the data are scaled from -0.35 to 0.7 with smoothing = 6.

The Differential Nuclease Sensitivity (DNS) values are calculated as the difference (Light digest normalized reads “minus” Heavy digest normalized reads) between light and heavy relative read coverage. As shown in Fig. 1, plotting the DNS profiles along a genomic region reveals areas with both positive (MNase Sensitive - blue) or negative (MNase Resistant - red) values. DNS-seq profiles were calculated in this way for four reference tissues (15 DAP endosperm, 1 mm terminal root tips, 1–2 cm earshoots, and 4–5 day old seedling coleoptilar nodes). The DNS-seq data processing pipeline provided in Supplemental file 2 describes the sequential computational steps required to produce UCSC genome-browser-ready data tracks from NGS paired-end Illumina reads.

**Fig. 2:**
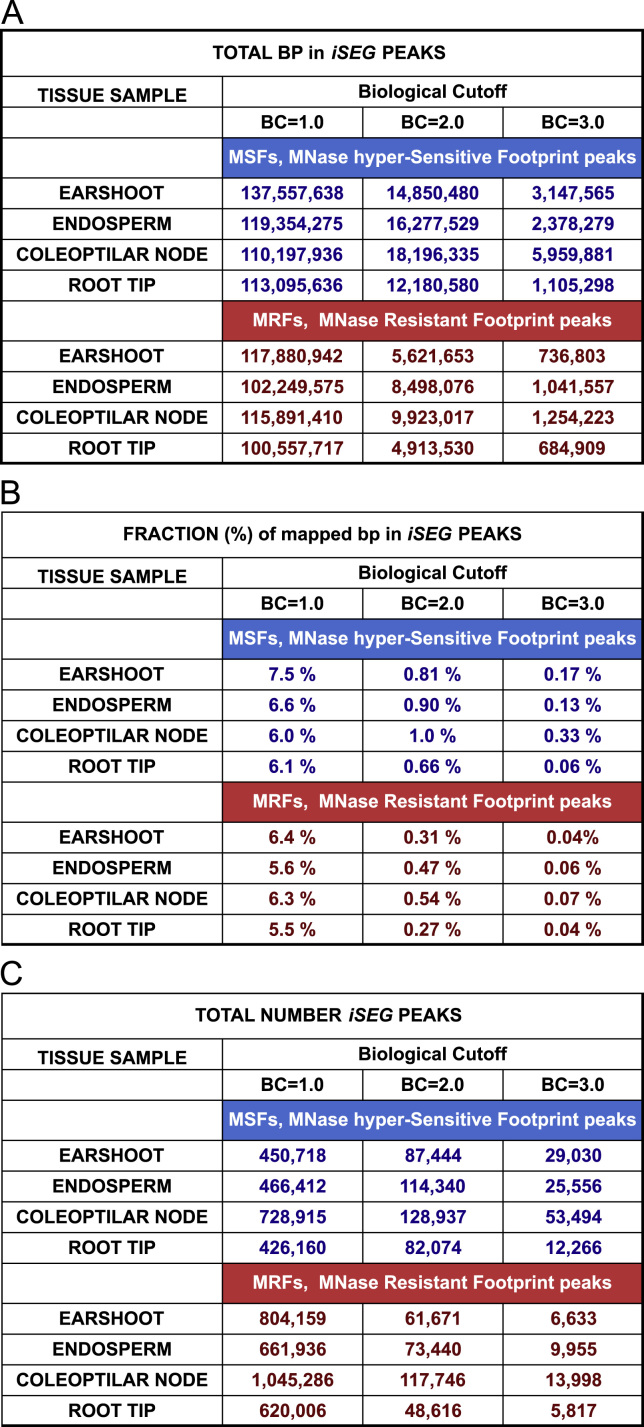
Summary statistics for iSeg peaks for the DNS-seq profiles of the four core reference tissues. Segments at three "biological cutoffs" (BC=1.0, 2.0, and 3.0) are tabulated separately for positive (MSF) and negative (MRF) peaks for each of the four tissues. Values summed over the whole genome are given for segment (A) total bp, (B) fraction of mapped bp, and (C) total number.

In order to delineate significant differences in global MNase-sensitivity, we segmented the profiles using the peak-calling program iSeg [4]. Descriptive statistics of these DNS-seq peak segments are shown in Fig. 2. The computational pipeline used to produce the iSeg peak calls is described in Supplemental file 3.

### 2.2 Plant materials

Maize (*Zea mays L*) B73 seeds were obtained from WF Thompson (NC State University, Raleigh, NC, USA, accession “27 FARMS”, bulked for NCSU, denoted B73^NC27^). Earshoot and endosperm tissues were collected from plants that were grown in the field at the Florida State University Mission Road Research Facility (Tallahassee, FL, USA). Tissue harvest for field-grown tissues was done at 9–11 A.M. by flash-freezing in liquid nitrogen. Coleoptilar nodes were collected from greenhouse-grown (King Greenhouse-room A, Biological Science Dept., FSU, Tallahassee, FL, USA) seedlings. Root tips were collected from lab-grown (WF Thompson Lab, NC State University, Raleigh, NC, USA) seedlings as described below.

### 2.3 Earshoot, 1–2 cm, Field-grown

Earshoots were harvested in the field at 9–11 A.M. and immediately frozen in liquid nitrogen. For each earshoot collected, stalks were cut at the base, the top or second from the top earshoot was removed, the husks were peeled off, the silks were gently but quickly rubbed off, the earshoot was cut at the base and measured. Earshoot samples were immediately frozen in liquid nitrogen, pooled by date of harvest, and transferred to -80°C freezers for storage.

### 2.4 Endosperm, 15 days after pollination, field-grown

Endosperm from self-pollinated ears was harvested at 15 days after pollination by manual microdissection in the field at 9–11 A.M. and immediately frozen in liquid nitrogen. For each ear, husks were removed, kernels detached, and the endosperm was separated from the embryo, scutellum, and pericarp. For each ear, only 10–20 kernels were quickly removed in order to limit the time elapsed between ear removal and tissue freezing to less than 5 min. The process was repeated for multiple ears during the 9am-11am time window for each day of harvest. Endosperm samples were immediately frozen in liquid nitrogen, pooled by date of harvest, and transferred to -80 °C freezers for storage.

### 2.5 Coleoptilar Node, 4–5 days after planting, 5 mm section of greenhouse-grown, spear stage seedlings

Coleoptilar nodes were harvested from greenhouse-grown seedlings 4–5 days after planting. Seeds were planted 2/3 deep in 2 in. of soil (“Maize Soil Mix”: per batch = 10 gallon Fafard #3 mix: 5 gal coarse sand, and 200 mL Osmocote fertilizer pellets (Scott׳s, 18.6.1)) spread into planting flats on greenhouse tables with natural plus supplemental lighting. The soil was well watered on day 1 and kept moist until harvest. Coleoptilar nodes were harvested between 10 AM and 12 PM on the day that “spears” showed above ground. Seedlings were quickly removed from the soil and first cut with a razor blade just above the seed. A 5 mm segment centered on the CN was then excised and immediately transferred to liquid nitrogen. Seedling removal and CN collection was done sequentially in order to limit the handling time to less than 1 min per plant. The CN samples were pooled by date of harvest, and transferred to −80 °C freezers for storage (Table 1).

**Table 1:** Sample information.

Harvest location	Tissue type	Biological replicate number	Digest level Heavy (H) or Light (L)	Technical replicate (a or b)	Read number (R1 or R2)
FSU (F)	coleoptilar node (CN)	1 or 2	L or H	N/A	R1 or R2
FSU (F)	endosperm (EN), 15 days after pollination	1 or 2	L or H	N/A	R1 or R2
FSU (F)	earshoot (ES), 1–2 cm	1 or 2	L or H	N/A	R1 or R2
FSU (F)	root tip (RT), 1 mm	1, 2, 3, or 4	L or H	a or b (for bioreps 1 and 2 only)	R1 or R2

In order to facilitate subsequent analysis, each data file (.fastq formatted) described in the NCBI SRA BioProject Accession HYPERLINK "https://www.ncbi.nlm.nih.gov/bioproject/PRJNA445708" \h PRJNA445708 can be named using the above schema to capture uniquely identifying information about each sample in the file name.

### 2.6 Root Tip, 3.5 days after imbibition, terminal 1 mm tips of primary and seminal roots

Root tips were harvested, fixed, and frozen from laboratory-grown seedlings using a modified protocol adapted from a technique developed to investigate plant DNA replication [5]. Seeds were imbibed in running water overnight, and germinated in sterile Magenta boxes (Sigma-Aldrich) containing a damp paper towel, and seedlings were grown under constant light at 28 °C for 72 h. The root tips were collected by cutting off the terminal 1 mm of primary and seminal root tips, fixing in 1% formaldehyde for 20 min, rinsing 3X in Buffer A [2], and flash freezing in liquid nitrogen. This fixed and frozen tissue, unlike the other tissues, was broken open via gentle brief polytron (speed 2–3, 15 s) of root tips from ~ 160 seedlings directly in MNase digestion buffer [2] followed by microfiltration through 50 μm Partec filter.

## 3 Methods

A general methodology for the isolation of plant nuclei, MNase digestion, and subsequent NGS library preparation are described in detail in supplemental file 1, “DNS Bench Protocol”.

Methodology for the processing of raw NGS data to browser-ready DNS profile data tracks are described in detail in supplemental file 2, “DNS Pipeline”.

Methodology for the segmentation of the DNS profiles are described in detail in supplemental file 3, “iSeg Pipeline”.
