# Supplementary material for: Chromatin structure profile data from DNS-seq: Differential nuclease sensitivity mapping of four reference tissues of B73 maize (Zea mays L)
Source: Data Brief. 2018 Aug 10;20:358–63. doi: 10.1016/j.dib.2018.08.015 (PMC6117953; doi:10.1016/j.dib.2018.08.015)
Supplement: Supplementary file 2 — Supplementary material [file mmc2.docx]

**Supplemental File 1**. DNS Bench Protocol

**Materials**

**0.5 M EGTA**: 0.5 M EGTA in ddH_2_O pH 8.0 (adjust with NaOH), sterilize with 0.2 µm filter.

**1:1 Phenol:chloroform pH 8.0**

**10% Paraformaldehyde**: 10% Paraformaldehyde in ddH_2_O. Prepare fresh and use in fume hood.

**10% SDS**:10% SDS in ddH_2_O, sterilize with 0.2 µm filter.

**10% Triton X-100:** dissolve 10% Triton X-100 in ddH_2_O overnight with constant mixing, sterilize with 0.2 µm filter.

**1000x Dithiothreitol (DTT):** 1.0 M DTT, 0.01 M Sodium acetate in ddH_2_O, sterilize with 0.2 µm filter. Aliquot and store at -80ºC.

**1000x Polyamines:** 0.15 M Spermine tetra HCl, 0.5 M Spermidine in ddH_2_O, sterilize with 0.2 µm filter. Aliquot and store at -80ºC.

**10X BFA salts:** 150 mM PIPES, 800 mM KCl, 200 mM NaCl, 20 mM EDTA, and 5 mM EGTA in ddH_2_O pH 6.8 (adjust with NaOH), sterilize with 0.2 µm filter. Aliquot and store at -80ºC.

150 µm Partec filters (Sysmex Partec)

**2.5 M Glycine**: 2.5 M Glycine in ddH_2_O, sterilize with 0.2 µm filter.

**20,000 U/mL MNase:** Dissolve 45,000 Units of lyophilized MNase in 2.25 mL MNase storage buffer.

**200 mM Phenanthroline:** 200 mM Phenanthroline in Isopropano**l**

**200 mM Phenylmethylsulfonyl fluoride (PMSF)**: 200 mM PMSF in Isopropanol.

**3M Sodium acetate**: 3 M Sodium acetate in ddH_2_O pH 5.2 (adjust with acetic acid), sterilize with 0.2 µm filter.

**5X Sorbitol:** 1.6 M Sorbitol in ddH_2_O, sterilize with 0.2 µm filter. Aliquot and store at -80ºC.

**DAPI:** 10 µg/mL DAPI in ddH_2_O

**Fixation Buffer:** 6 mL ddH_2_O, 1 mL 10X Buffer A Salts, 2 mL 5X sorbitol, 10 µL 1000X DTT, 10 µL 1000X polyamines , 10 µL 200 mM PMSF, 10 µL 200 mM phenanthroline, 1 mL 10% paraformaldehyde. Prepare immediately before use in a fume hood and directly dispense after mixing.

**Miracloth** (EMD Millipore)

**MNase Digestion Buffer (MDB)**: 50 mM HEPES pH 7.6, 12.5% Glycerol, 25 mM KCl, 4 mM MgCl_2_, 1 mM CaCl_2_, sterilize with 0.2 µm filter.

**MNase Digestion Buffer + 1% Triton X-100 (MDBT)**: MDB made to include 1% Triton X-100.

**MNase Storage Buffer:** 10 mM Tris-HCl, 50 mM NaCl, 1mM EDTA, 50% Glycerol, pH 7.5, sterilize with 0.2 µm filter.

**NEBNext Ultra II DNA Library Prep kit** (New England Biolabs)

**Proteinase K**: 20 mg/mL Proteinase K in Tris-HCl pH 8.0, 50% Glycerol

**TE**: 10 mM Tris-HCl pH 7.6, 1 mM EDTA-NaOH pH 8.0, autoclave and pass through 0.2 µm filter.

**TER**: 10 mM Tris-HCl pH 7.6, 1 mM EDTA-NaOH pH 8.0, 40 µg/mL RNase A, autoclave then 0.2 µm filter prior to addition of RNAse A.

**TR:** 10 mM Tris-HCl pH 7.6, 40 µg/mL RNase A, autoclave, then 0.2 µm filter prior to addition of RNase A.

**TT-20 Buffer;** 10 mM Tris-HCl pH 8, 0.2% Tween-20, autoclave, then 0.2 µm filter prior to addition of Tween-20.

**Specialist Equipment:**

Agencourt AMPure XP beads (Beckman Coulter)

DynaMag-2 Magnetic 1.5 mL Tube rack (Thermo Fisher Scientific)

“Qubit” Dye-based DNA concentration fluorometer (Thermo Fisher Scientific)

Agilent 2100 Bioanalyzer for analysis of DNA fragment size and concentration

High sensitivity DNA kit (Agilent)

Polytron PT 10-35 tissue disruptor (Kinematica)

**==============================================================**

**Methods for 1 Gram of Tissue**

(or for ~1 mL Ground Tissue Powder)

**==============================================================**

**1 Harvest, Fixation, and Breakage of Plant Tissues**

1. Harvest tissue as rapidly as practical^^[[1]](#footnote-1)^^ by freezing in liquid nitrogen^^[[2]](#footnote-2)^^.
2. Grind tissue to compete fine powder under liquid nitrogen without thawing.^^[[3]](#footnote-3)^^
3. Set aside at least 10% frozen, unfixed tissue for optional future analyses, such as RNA-seq or proteomics.
4. Distribute ~1.0 g frozen tissue powder per 50 mL conical^^[[4]](#footnote-4)^^.
5. Fix the tissue by addition of 10 mL of fresh-made Fixation Buffer^^[[5]](#footnote-5)^^ containing 1% paraformaldehyde. Allow fixation to proceed for 10 minutes at RT with constant gentle mixing^^[[6]](#footnote-6)^^.
6. Stop fixation reaction by addition of 0.1 vol (~1 mL) of 2.5 M glycine to achieve a final concentration of 250 mM glycine, with constant, gentle mixing at RT for 5 minutes.
7. Remove ~50 µL of fixed cells for Microscopic Check 1 “MC1” and hold at 4ºC.
8. Dilute fixed tissue into MDB^^[[7]](#footnote-7)^^ to a final volume of 45 mL.
9. Concentrate fixed tissue by centrifugation at 1500 rcf for 5 minutes at RT.
10. Resuspend fixed tissue in 4 mL MDB with 1% Triton X-100 (MDBT).
11. Remove ~50 µL of fixed cells for Microscopic Check 2 “MC2” and hold at 4ºC or DAPI stain and check at this point to confirm tissue breakage and nuclei release^^[[8]](#footnote-8)^^.
12. (Optional) If warranted from MC2, further mechanically disrupt tissues with a Polytron for 1 minute to fully homogenize fixed tissue to liberate nuclei^^[[9]](#footnote-9)^^.
13. Incubate at RT with constant gentle mixing by inversion for 5 minutes.
14. Remove ~50 µL of fixed cells for Microscopic Check 3 “MC3” and hold at 4ºC to confirm presence of nuclei or nuclei-containing cell fragments.

**2 Isolation of Formaldehyde-Fixed Nuclei**

1. Filter lysate through a 150 µm partec filter (or through a funnel lined with 1 layer of miracloth) into a new 15 mL polypropylene conical tube.^^[[10]](#footnote-10)^^
2. Split the suspension equally between two new 15 mL centrifuge tubes.^^[[11]](#footnote-11)^^
3. Dilute Percoll to a final concentration of 60% v/v in MDB.
   1. Combine 2.4 mL Percoll and 1.6 mL MDB in a new tube for each aliquot of lysate. (2 aliquots/g input tissue)^^[[12]](#footnote-12)^^
4. Using a 10 mL serological pipette, slowly add 4 mL 60% percoll to the bottom of each lysate-containing conical, producing two distinct phases with a visible interface^^[[13]](#footnote-13)^^.
5. Centrifuge at 3000 rcf for 15 minutes at 4ºC with slow acceleration/deceleration.^^[[14]](#footnote-14)^^
6. Aspirate off most of the upper phase, taking care not to disturb the interface. Leave ~2 mL of the upper phase.^^[[15]](#footnote-15)^^
7. Collect nuclei at the interface between the low density lysate and the percoll layer with a 10 mL serological pipette into a single new 15 mL polypropylene conical tube.^^[[16]](#footnote-16)^^
8. Dilute nuclei into a final volume of 15 mL MDB.^^[[17]](#footnote-17)^,^[[18]](#footnote-18)^^
9. Pellet nuclei by centrifugation at 2000 rcf for 15 minutes at 4ºC.
10. Discard the supernatant and resuspend nuclei in 15 mL ice cold MDB.^^[[19]](#footnote-19)^^
11. Pellet nuclei by centrifugation at 2000 rcf for 15 minutes at 4ºC.
12. Resuspend nuclei in 2.2 mL ice-cold MDBT.^^[[20]](#footnote-20)^^
13. Aliquot 500 µL nuclei into each of four 1.5 mL screwcap tubes.^^[[21]](#footnote-21)^^
14. Keep the remaining nuclei as microscopic check “MC4” to confirm recovery of intact nuclei and removal of cellular debris.^^[[22]](#footnote-22)^^
15. Flash freeze all four aliquots of isolated nuclei in liquid nitrogen for future use or long term storage at -80ºC.^^[[23]](#footnote-23)^^
16. End of prep microscopy check: Examine MC1 - MC4 to confirm the quality, approximate quantity, and purity of the nuclei, preparing slides as follows.
    1. For each 50 µL aliquot, add 5 uL 10 µg/mL DAPI (~ 1µg/mL DAPI final).
    2. To each slide, add 5 uL of DAPI-stained nuclei followed by 10 uL mounting medium. Mix by stirring with the end of pipette, and seal under a glass coverslip.
    3. Examine nuclei by epifluorescence microscopy with a 60X objective lense.^^[[24]](#footnote-24)^,^[[25]](#footnote-25)^,^[[26]](#footnote-26)^,^[[27]](#footnote-27)^^

**3 Analytical MNase Titration; wide-range digestions**

1. Thaw 1 (tube #1 of 4) of the 500 µL nuclei aliquots at RT, gently resuspend.
2. Aliquot 60 uL into each of eight 1.5 mL screw-cap centrifuge tubes.
3. Prewarm the eight tubes of nuclei in 37ºC heat block while preparing MNase series.
4. Prepare enzyme dilutions by 3-fold serial dilution starting with 7,300 U/mL MNase in MDB.
   1. To make the initial stock of 7,300 U/mL, add 7.3 µL 20,000 U/mL MNase stock to 12.7 µL MDB and briefly vortex before briefly centrifuging.
   2. For the 3-fold serial dilutions, transfer 5 µL of enzyme into a 10 µL diluent (MDB) and briefly vortex before briefly centrifuging.
   3. Repeat to produce 7 enzyme tubes with U/mL concentrations of 7,300; 2,400; 810; 270; 90; 30; plus one last tube for 0 u/mL (MDB alone).
5. Digest nuclei at 37ºC for 15 minutes by rapid addition and quick, thorough mixing of 6.7 µL from enzyme dilution tubes to seven of the eight 60 µL nuclei aliquots.^^[[28]](#footnote-28)^^ Add 6.7 µL of MDB to the final tube.^^[[29]](#footnote-29)^^
6. Stop reactions by rapid addition of 5µL 0.5M EGTA-NaOH (8.0)
7. Add decrosslinking ingredients: 390 µL water, 50 µL 10% SDS, and 5 µL 20 mg/mL proteinase K. Briefly vortex to mix.
8. Incubate at 65ºC overnight (12-16h) to reverse the formaldehyde crosslinks.

**4 Purification of DNA from Analytical MNase Titration.^^[[30]](#footnote-30)^^**

1. Prewarm the elution buffer (TR) at 37ºC.
2. Add 600 µL Zymo ChIP Binding Buffer (“Zymo” here refers to cat. D5205 ChIP DNA cleanup kit) to each decrosslinked sample (containing ~ 517 uL) and mix by vortexing.
3. Transfer mixture to a Zymo column in a new Zymo collection tube and microcentrifuge at top speed (~20,000 rcf) for 30 seconds at RT.
4. Discard flow-through, add 200 µL Zymo wash buffer to the column, and microcentrifuge at top speed for 30 seconds at RT.
5. Repeat the previous wash step once more and discard the flow-though.
6. Dry column by microcentrifugation at top speed for an additional 30 seconds at RT.
7. Elute DNA in TR by transferring the column to a new 1.5 mL tube, adding 30 µL of 37ºC TR and incubate for 1 min, followed by microcentrifugation at top speed for 30 seconds at RT.
8. Quantify nucleic acids by UV spectroscopy and make gel-ready samples with matched total nucleic acids (select an amount in the 100 - 500 ng/lane range).
9. Run analytical gel using 1% Agarose/TBE gel at 4-10 V/cm.
10. Visualize DNA by routine EtBr staining and photography.
11. Analyze digestion patterns to choose the desired degree(s) of digestion for the pair of preparative digests, Heavy and Light.^^[[31]](#footnote-31)^^ Digest level selection is one of the most important steps in DNS chromatin profiling, as discussed in the notes.

**5 Preparative MNase Digests**

1. Thaw one (“2 of 4”) 500 µL aliquot of frozen, fixed nuclei at RT.
2. Split nuclei into 2 screwcap 1.5mL centrifuge tubes, 100 µL for Heavy, 400 µL for Light.
3. Prewarm nuclei to 37ºC on a heat block.
4. Prepare the two MNase 10X stocks in MDB on the basis of selected concentrations for Heavy and Light from the analytical MNase titration.^^[[32]](#footnote-32)^^
5. Add in 0.1 Volume^^[[33]](#footnote-33)^^ of each MNase 10X stock to the respective 37ºC nuclei by rapid addition with quick and thorough mixing.
6. Incubate MNase digests at 37ºC for 15 minutes.
7. Stop reactions by adding 0.1 Volume 0.5 M EGTA-NaOH (8.0).
8. Add 50 µL 10% SDS and 5 µL 20 mg/mL proteinase K. Briefly vortex to mix.
9. Dilute each sample to a final volume of 500 µL with ddH_2_O.
10. Reverse the formaldehyde crosslinks by incubation overnight (12-16h) at 65ºC.

**6 Purification of DNA from Preparative Digests**^^[[34]](#footnote-34)^^

1. Remove tubes from 65ºC incubator. Allow samples to cool to RT.
2. Phase extract the DNA with 1:1 phenol(pH 8):chloroform^^[[35]](#footnote-35)^^.
   1. Add ~1 Volume (600 µL) 1:1 Phenol:Chloroform (pH 8) to each tube.
   2. Vortex for 1 minute.
   3. Centrifuge at top speed (~20,000 rcf) for 10 minutes at RT with slow deceleration.
   4. Transfer top (aqueous) phase containing DNA to a new 1.5 mL screwcap tube. Discard the lower, organic phase into the appropriate hazardous waste.
3. Phase extract again with 1:1 phenol:chloroform (pH 8.0) as above.
4. Phase extract again with chloroform only, as above.
5. Precipitate the DNA by addition of 0.1 Volume of 3 M sodium acetate (pH 5.2), 1 µL 25 mg/mL LPA^^[[36]](#footnote-36)^^, and then 2 Volumes of 100% -20ºC ethanol and briefly vortex to mix.
6. Incubate at -20ºC for at least 30 minutes.
7. Centrifuge at top speed (~ 20,000 rcf) 20,817 rcf for 30 minutes at 4ºC.
8. Discard supernatant and wash the pellet with 1 mL 70% -20ºC ethanol.
9. Centrifuge at top speed (~ 20,000 rcf) 20,817 rcf for 10 minutes at 4ºC.
10. Discard supernatant and air dry the DNA pellet.
11. Resuspend DNA in 100 µL 37ºC TER, and incubate for 1h at RT to remove RNA.
12. Repeat the Ethanol precipitation described steps 6-11, but omitting LPA.
13. Resuspend final dried DNA pellet in 50 µL 37ºC TE.
14. Quantify DNA by spectrophotometry.
15. Analyze 50-100 ng DNA on a 1% agarose gel to verify successful Heavy and Light digests. Store purified DNA at -80ºC until further use.
16. Carry out the fragment size selection using the guidelines in Table 1. The Ampure bead values are given on the basis of genomic DNA fragment sizes before library preparation. It is also possible to make the library from the entire digest, and then size select afterwards, making the necessary adjustments for increase in fragment lengths following adaptor ligation and indexing PCR.

Table 1. Guidelines for size-selection relative to experimental objectives.

| **Standard Digest Levels for DNS-seq** | | | | | |
| --- | --- | --- | --- | --- | --- |
| Digest level | Target  footprint | Fragment Size Cutoff | | Experimental Objective | maize sequencing depth |
|  |  | Upper, 1st Ampure Beads | Lower, 2nd Ampure Beads |  |  |
| **Heavy** | mononucleosome | 0.6X  (< 200 bp) | 0.8X  (> 130 bp) | stable nucleosomes | 160M clusters |
| **Light** | mononucleosomes and sub-nucleosome sized particles | 0.6X  (< 200 bp) | 1.5X  (> 30bp) | open chromatin, fragile nucleosomes^a^ and small particles^b^ | 160M clusters^c^ |
| ***Variations on Size-Selection for Specialized Applications** | | | | | |
| Digest level | Target  footprint | Fragment Size Cutoff | | Experimental Objective | maize sequencing depth |
|  |  | Upper, 1st Ampure Beads | Lower, 2nd Ampure Beads |  |  |
| **Light** | mononucleosome | 0.6X | *0.8X | open chromatin, fragile nucleosomes | 160M clusters |
| **Light** | Sub-nucleosome sized Particles | *0.8X | 1.5X | open chromatin, small particles | 20M clusters |

Table Notes:

1. “fragile nucleosomes” refers to nucleosome-sized fragments with higher coverage in Light vs. Heavy digests (a type of MSF as described by Vera et al., 2014).
2. “small particles” refers to subnucleosome-sized fragments such as those from TF-bound sites. These fragments will closely resemble DNase I HS or ATAC seq-like fragments.
3. Seq Depth: 80M fragments / Gb / replicate (= 160M clusters/fragments for Z. mays)

**7 Prepare and check NGS sequencing libraries:**

1. Construct NGS libraries according to manufacturer’s directions. ^^[[37]](#footnote-37)^^
2. Assess DNA yield with Qubit fluorometric quantitation.
3. Assess fragment size distribution with an Agilent Bioanalyzer HS DNA chip.
4. Libraries are ready for final qPCR quantitation, pooling, verification of pooled quantity by qPCR and bioanalyzer, and Illumina NGS.

1. Tissues should be rapidly harvested under closely matched conditions, including time of day. Tissues should be flash-frozen in liquid nitrogen within 10-60 seconds of harvest. These flash-frozen tissues can be stored at -80ºC indefinitely. [↑](#footnote-ref-1)
2. Liquid nitrogen is a potentially hazardous cryogen. Protective hand and eye wear must be worn at all times when handling liquid nitrogen. Non-reinforced containers (such as polypropylene tubes) of liquid nitrogen cannot be sealed before the nitrogen evaporates, or it might build pressure and explode the sealed container. [↑](#footnote-ref-2)
3. Materials and equipment necessary for this step should be labeled, arranged, and pre-chilled prior to removing frozen tissues from -80ºC storage. Vent (1) 50 mL polypropylene conical tube / frozen sample by puncturing three (3) holes in the lid of each tube with a 28 gauge syringe to prevent explosive pressure from building above liquid nitrogen. Prechill all conical tubes on dry ice. Pre-chill mortar and pestle by filling mortar with liquid nitrogen with pestle inside. Transfer tissue directly into a liquid nitrogen-filled mortar and grind to a fine powder under nitrogen, slowly adding liquid nitrogen as needed to prevent thawing. [↑](#footnote-ref-3)
4. Tissue must be kept on dry ice until all nitrogen has *completely* evaporated. Vented lids can then be replaced with new, intact lids. Tissue aliquots may be stored at -80ºC long-term or held on dry ice for immediate processing. [↑](#footnote-ref-4)
5. 10 mL Fixation Buffer per ~1g tissue aliquot must be prepared fresh at room temperature just before use from constituent solutions (see materials). [↑](#footnote-ref-5)
6. Complete fixation buffer should be mixed with a 10 mL pipette and directly dispensed to a ~1g aliquot of frozen tissue powder. Aggregates of frozen tissue must be dispersed prior to incubation at room temperature to ensure complete fixation of powdered tissue. Failure to disperse tissue aggregates during resuspension will increase the frequency of nuclei with abnormal morphology such as ruptured nuclei or degraded chromatin. Once the tissue is resuspended, place on rotary shaker, nutator, or rotating mixer. [↑](#footnote-ref-6)
7. Dilution to ~50 mL decreases the density of the sorbitol-containing fixation buffer to allow fixed cells to pellet during centrifugation for maximum recovery of fixed cells prior to lysis. [↑](#footnote-ref-7)
8. Qualitative examination of DAPI-stained nuclei at 60x magnification at this step is sufficient to confirm successful lysis of fixed cells. If very large aggregates of cellular debris with few intact nuclei are present, it may be necessary to subject the sample to further mechanical disruption (see optional step 1.12). Quantitative examination of DAPI-stained, fixed nuclei can be performed at this stage using a hemacytometer or similar equipment. In our experience, observation of three or more intact nuclei per field of view at 60x magnification is indicative of a sufficient yield, even if cellular debris is evident. [↑](#footnote-ref-8)
9. Meristematic tissues do not usually require further mechanical disruption after thorough resuspension in MDBT. [↑](#footnote-ref-9)
10. For some tissues, successful MNase Digests have been produced without percoll-purification steps (2.2-7). If highly purified nuclei are obtained at MC2 or MC3, or if nuclei are trapped in cell fragments, the percoll purification can be omitted. [↑](#footnote-ref-10)
11. For a 4g input, split the suspension equally between two new 50 mL centrifuge tubes. [↑](#footnote-ref-11)
12. For a 4g input, combine 6 mL Percoll and 4 mL MDB in a new tube for each aliquot of lysate. (2 aliquots/4 g input tissue) [↑](#footnote-ref-12)
13. For a 4g input, add 10 mL 60% percoll to the bottom of each lysate-containing conical. [↑](#footnote-ref-13)
14. Slow acceleration at this step minimizes the chance of disturbing the interface between the sample in MDBT and the heavier percoll. [↑](#footnote-ref-14)
15. For a 4g input, leave ~5 mL of the upper phase. [↑](#footnote-ref-15)
16. For a 4g input, combine nuclei in a new 50 mL polypropylene conical tube. [↑](#footnote-ref-16)
17. For a 4g input, dilute the nuclei suspension to a final volume of 50 mL with MDB. [↑](#footnote-ref-17)
18. Dilution of nuclei-containing aspirate is necessary to decrease the density of the solution to allow for centrifugal sedimentation of nuclei. [↑](#footnote-ref-18)
19. For a 4g input, dilute nuclei to 50 mL. [↑](#footnote-ref-19)
20. For a 4g input, resuspend nuclei in 4 mL ice-cold MDB containing 1% Triton X-100. [↑](#footnote-ref-20)
21. Label tubes “1 of 4” through “4 of 4”. The first tube will be used for the analytical titration, the second for the preparative digests, and the last two as extras in case needed. [↑](#footnote-ref-21)
22. After this point, the protocols for a 4g and 1g preps are identical. [↑](#footnote-ref-22)
23. Flash freeze all aliquots to prevent variation between samples due to one freeze-thaw cycle. [↑](#footnote-ref-23)
24. MC1 (5µL of 11 mL)- observe quenched, fixed cells. Large tissue aggregates (of >20 cells) indicate incomplete tissue disruption and could explain low final nuclei yield or “smear” following MNase titration. [↑](#footnote-ref-24)
25. MC2 (5 uL of 4 mL lysate)- observe lysis of fixed cells. Should observe cellular debris, wall fragments, etc in addition to intact, fixed nuclei, and can include nuclei in broken cell fragments. [↑](#footnote-ref-25)
26. MC3 (5 uL of 4 mL homogenized lysate) - observe lysis of fixed cells. Should observe cellular debris, wall fragments, etc in addition to intact, fixed nuclei, and can still include nuclei in broken cell fragments. [↑](#footnote-ref-26)
27. For MC4 (5 uL of 2.2 mL purified nuclei) - observe isolated, fixed nuclei, and some residual cellular debris. Observation with 40X-60X lense can be used to estimate “good yields” suitable for proceeding. Here, good yields refer to the ability to readily detect nuclei when scanning around and looking through the eyepiece. [↑](#footnote-ref-27)
28. MNase titrations must be performed in such a way as to minimize the amount of time between addition of reagents to multiple samples, as small variations in reaction duration can impact degree of digestion of fixed chromatin. [↑](#footnote-ref-28)
29. The final tube serves as a MNase-negative control for non-MNase DNA degradation during purification and nuclease contamination. [↑](#footnote-ref-29)
30. PCR or ChIP cleanup kits are used for analytical gels. We use the “ChIP DNA Clean & Concentrator” kit (Zymo, cat. D5205) with modifications. [↑](#footnote-ref-30)
31. The highest MNase concentration with a remaining single band of genomic large fragments is designated the “light” digest condition. The lowest MNase concentration with the vast majority of fragments in the mononucleosome size range (~150bp) is designated the “heavy” digest condition. [↑](#footnote-ref-31)
32. Example: If the final MNase concentration in the reaction chosen as the “light” digestion condition is 3 U/mL, a sufficient volume of 10X “light” MNase for addition to 400 µL nuclei (at least 44 µL) in MDB must be prepared at 30 U/mL. [↑](#footnote-ref-32)
33. For 100 and 400 µL nuclei, 0.1 Volume refers to 10 µL for 10X Heavy and 40 µL 10X Light, respectively. [↑](#footnote-ref-33)
34. If samples are not already in screw cap tubes with air-tight gasket seals, transfer samples to these tubes. Organic solvents used in the extraction of hydrophobic proteins and nuclear debris have significant vapor pressure at room temperature and atmospheric pressure. Use of improper containers at this step may result in sample loss due to tube-lid failure. [↑](#footnote-ref-34)
35. Halogenated and non-halogenated organic solvents must be disposed of according to all institutional and governmental regulations. [↑](#footnote-ref-35)
36. Co-precipitation carriers such as Linear Polyacrylamide are used as an inert carrier for DNA precipitations to increase efficient recovery from low concentration samples and to increase visibility of the resulting DNA pellet. [↑](#footnote-ref-36)
37. For DNS-seq libraries, we use 200-500 ng DNA per library, using NEBNext® Ultra™ II DNA Library Prep (Cat. E7103S).
     [↑](#footnote-ref-37)
