# Supplementary material for: Chromatin structure profile data from DNS-seq: Differential nuclease sensitivity mapping of four reference tissues of B73 maize (Zea mays L)
Source: Data Brief. 2018 Aug 10;20:358–63. doi: 10.1016/j.dib.2018.08.015 (PMC6117953; doi:10.1016/j.dib.2018.08.015)
Supplement: Supplementary file 3 — Supplementary material [file mmc3.docx]

# **Supplemental File 2**. DNS-seq Data Processing Pipeline

*Comments in italics, from DL Vera, 2018.*

Linux commands follow "**$**"

R commands follow "**>**"

# 1. Setup

## 1.1 Installation of command-line programs in bash on your linux box

*1. install cutadapt*

**$** pip install --user cutadapt

*2. setup directory to store executables and define PATH*

**$** mkdir -p ~/usr/bin

**$** echo 'export PATH=${HOME}/usr/bin:${HOME}/.local/bin:${HOME}/usr/opt/FastQC:$PATH' >> ~/.bashrc

**$** source ~/.bashrc

*3. install bedtools*

**$** cd ~ && wget -O-

https://github.com/arq5x/bedtools2/releases/download/v2.26.0/bedtools-2.26.0.tar.gz | tar zx && cd bedtools-2.26.0 && make && cp bin/* ~/usr/bin/

*4. install kent source utilities*

**$** cd ~ && wget -P kent -r -np -nd -e robots=off -R 'html' http://hgdownload.soe.ucsc.edu/admin/exe/linux.x86_64/ && rm kent/index* && chmod -R 755 kent && chmod +x kent/* && find kent/ -maxdepth 1 -perm -111 -type f | grep -v install | xargs -I'{}' cp '{}' ~/usr/bin/

*5. install FastQC*

**$** cd ~ && wget --no-check-certificate https://www.bioinformatics.babraham.ac.uk/projects/fastqc/fastqc_v0.11.5.zip && unzip fastqc_v0.11.5.zip && chmod +x FastQC/fastqc && mkdir ~/usr/opt/ && mv FastQC ~/usr/opt/

*6. install bowtie2*

**$** wget --no-check-certificate -O bowtie2.zip "https://downloads.sourceforge.net/project/bowtie-bio/bowtie2/2.3.1/bowtie2-2.3.1-linux-x86_64.zip?r=https%3A%2F%2Fsourceforge.net%2Fprojects%2Fbowtie-bio%2Ffiles%2Fbowtie2%2F2.3.1&ts=1491582173&use_mirror=iweb"

*7. unzip bowtie2.zip and copy to usr/bin/*

**$** cp bowtie2-2.3.1/bowtie2 bowtie2-2.3.1/bowtie2-* ~/usr/bin/

1.2 Installation of R packages

*1. open R*

**$** R

*2. install devtools and daniel’s packages*

**>** install.packages("devtools")

**>** install.packages("gplots")

**>** devtools::install_github("dvera/conifur")
**>** devtools::install_github("dvera/converge")
**>** devtools::install_github("dvera/gyro")
**>** devtools::install_github("dvera/travis")

**>** q()

**>** y

# 2. DNS-seq pipeline

## 2.1 Align data and generate fragment bed files

*This pipeline assumes you already have a bowtie2 index. Make sure the index you use for bowtie, the genome browser you will use, and the chrom sizes file you create all have the same chromosome names and are for the same build (genome version).*

*1. Make a chrom sizes file, a tab-separated file that contains the chromosome name in the first column and the chromosome size in the second column (no commas).*

*2. navigate to the directory with your fastq files and open R.*

**$** cd /path/to/your/files

**$** R

*3. load travis and set threads*

**>** library(travis)

**>** options(threads=detectCores())

**>** options(verbose=T)

*4. Define the path to chrom sizes file and sort it*

**>** chromsizes="/path/to/chrom.sizes"

**>** bedSort(chromsizes)

*5. define fastq files (ok if gzipped)*

**>** f=files("*.fastq")

*6. run fastqc*

**>** fastqc(f)

*7. trim adapters*

**>** ft=cutadapt(f)

*8. define R1 and R2 adapter-clipped fastq files*

**>** r1=files("*_R1_*_clip.fastq")

**>** r2=files("*_R2_*_clip.fastq")

*9. align reads with bowtie2 (replace /path/to/index/prefix with the path to your bowtie2 index)*

**>** s=bowtie2(r1,"/path/to/index/prefix",r2,maxInsertSize=1000)

*10. convert sam to bam,*

*# keeping alignments with quality >=20*

**>** bams=samtoolsView(s,minQual=20)

*11. remove duplicates (Optional)*

**>** bams=samtoolsRmdup(bams)

*12. convert bam to fragment bed files*

**>** beds=bamToBed(bams,paired=T)

*13. examine fragment size distribution*

**>** bedHist(beds,xlims=c(0,200),dens=F,brks=100)

## 2.3 Parse bed files by fragment size and create fragment density bigWigs in 20 bp windows

*1. parse fragments by length*

**>** bpl=bedParseLengths(beds,c(0,130,260))

**>** bpl=unlist(bpl)

*2. calculate fragment densities.*

**>** allbeds=files("*.bed")

**>** bgs=bedtoolsGenomeCov(allbeds,chromsizes)

*3. unify the bedgraphs so they have the same coordinates*

**>** ubgs=bgUnify(bgs,filler=0,discardUnshared=F)

*4. define light and heavy unified bedGraphs. Assumes light-digest files have an L_ in their names, and heavy-digest have H_*

**>** l=files("*L_*unified.bg")

**>** h=files("*H_*unified.bg")

**>** allbeds=files("*.bed")

**>** w=bedtoolsMakeWindows(chromsizes,20,genome=T)

**>** bgs=bedtoolsCoverage(allbeds,w)

**>** bgs

*5. make sure only non-length-parsed data is selected. if not, subset bgs to only contain one fragment size class*

**>** qbgs=bgQuantileNorm(bgs)

**>** l=files("*L_*qnorm.bg")

**>** h=files("*H_*qnorm.bg")

*6. check to see if light and heavy are paired properly*

**>** data.frame(l,h)

*7. calculate difference between light and heavy*

**>** dbgs=bgOps(l,"difference",h,pattern="L_",replacement="D_")

*8. convert bedGraph files to bigWig*

**>** bws=bedGraphToBigWig(c(l,h,dbgs),chromsizes)

*9. The resulting bigWig files can be used on most genome browsers*
