# Supplementary material for: Chromatin structure profile data from DNS-seq: Differential nuclease sensitivity mapping of four reference tissues of B73 maize (Zea mays L)
Source: Data Brief. 2018 Aug 10;20:358–63. doi: 10.1016/j.dib.2018.08.015 (PMC6117953; doi:10.1016/j.dib.2018.08.015)
Supplement: Supplementary file 4 — Supplementary material [file mmc4.docx]

**Supplemental File 3**. iSeg Pipeline for DNS-seq positive and negative peak calling.

1. Get executable *iSeg* program and t-statistics list
   1. *iSeg*: <http://ani.stat.fsu.edu/~jinfeng/programs/iseg>
   2. T-statistics list: [note: this table will be hard-coded into future versions of *iSeg*]
      <https://www.bio.fsu.edu/bass/hwbtrax/nuprimeDataShare/iseg.dir/MeanCut.csv>
2. Input format

Each line in the input file must has four tab-separated entries: ***chrom***, ***chromStart***,

***chromEnd***, ***dataValue***. ***chrom*** must be integers. No characters or strings are allowed.


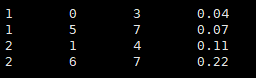


Figure 1. Example of input with two chromosomes, each with two regions and values.

1. Global ***sd*** (standard deviation) and ***mad*** (median absolute deviation)

Global ***sd*** and ***mad*** are computed from pooled and expanded data of all chromosome

by the following steps:

1. Expand data

The data must be expanded first based on the input data and the length of

Chromosome. Assuming the length of both chromosome 1 and 2 are 10, the

expanded data in Figure 1 will be:

[0.04, 0.04, 0.04, 0, 0, 0.07, 0.07, 0, 0, 0] & [0, 0.11, 0.11, 0.11, 0, 0.22, 0, 0, 0, 0]

1. Pool all chromosomes

Get pooled data by concatenating the expanded data of all chromosomes. The

pooled data in Figure 1 will be:

[0.04, 0.04, 0.04, 0, 0, 0.07, 0.07, 0, 0, 0, 0, 0.11, 0.11, 0.11, 0, 0.22, 0, 0, 0, 0]

1. X0 mode

Remove 0s from the pooled data. The data in Figure 1 will become:

[0.04, 0.04, 0.04, 0.07, 0.07, 0.11, 0.11, 0.11, 0.22]

1. Use the data resulted from c) to compute *sd* and *mad*. *mad* is defined as:

median(abs(data - median(data)))

1. Command for running iSeg

./iseg -p MeanCut.csv -sig 1e-3 -fdr 0.05 -bc X.X -minwl 20 -maxwl 100 -d INPUT.bg -of INPUT_x0_bcX.X.txt -ctp -cz -m 0 -mad *mad* -sd *sd*

Parameters description:

1. -sig: Significance cutoff for detecting significant segments.
2. -fdr: Cutoff for false discovery rate (FDR) control.
3. -bc: Biological cutoff value
4. -minwl: Minimum window length, the *W_min_* in iSeg paper.
5. -minwl: Maximum window length, the *W_max_* in iSeg paper.
6. -d: The file name of input data.
7. -of: The file name of iSeg results, which has six tab-separated columns: ***chrom***, ***SegmentStart***, ***SegmentEnd***, ***meanHeight***, ***tStatistics***, ***pValue***.
8. -ctp: Specify to compute true p-value from t-statistics.
9. -cz: Specify to compute z-statistics.
10. -m: 1 (the default value) indicates that to merge two adjacent significant segments; 0 indicates not to merge.
11. Merge adjacent segments with 1 base spacing (Fuse mode)

In the iSeg results output file, merge two adjacent segments with 1 base spacing if

1. ***chrom_1_*** equals to ***chrom_2_*** , and
2. (***SegmentEnd_1_ + 1***) equals to ***SegmentStart_2_*** , and
3. ***meanHeight_1_*** and ***meanHeight_2_*** have the same sign.

Update the t-statistics and p-value of the merged segment. Repeat this step until no

segments can be merged.

1. Use the first three columns of the iSeg results file to generate a BED file.

1. Convert BED file to bigBED using *bedSort* and *bedToBigBed* from [UCSC Genome Browser software](http://hgdownload.soe.ucsc.edu/admin/exe/).
2. Side notes
3. It is recommended to run iSeg on each chromosome separately to avoid crash due to memory insufficiency.
4. Narrowing down window length by tuning -minwl and -maxwl can reduce memory usage.
